# Costs Analysis of a Population Level Rabies Control Programme in Tamil Nadu, India

**DOI:** 10.1371/journal.pntd.0002721

**Published:** 2014-02-27

**Authors:** Syed Shahid Abbas, Manish Kakkar, Elizabeth Tacket Rogawski, on behalf of the Roadmap to Combat Zoonoses in India (RCZI) initiative

**Affiliations:** Public Health Foundation of India, Vasant Kunj, New Delhi, India; The Global Alliance for Rabies Control, United States of America

## Abstract

The study aimed to determine costs to the state government of implementing different interventions for controlling rabies among the entire human and animal populations of Tamil Nadu. This built upon an earlier assessment of Tamil Nadu's efforts to control rabies. Anti-rabies vaccines were made available at all health facilities. Costs were estimated for five different combinations of animal and human interventions using an activity-based costing approach from the provider perspective. Disease and population data were sourced from the state surveillance data, human census and livestock census. Program costs were extrapolated from official documents. All capital costs were depreciated to estimate annualized costs. All costs were inflated to 2012 Rupees. Sensitivity analysis was conducted across all major cost centres to assess their relative impact on program costs. It was found that the annual costs of providing Anti-rabies vaccine alone and in combination with Immunoglobulins was $0.7 million (Rs 36 million) and $2.2 million (Rs 119 million), respectively. For animal sector interventions, the annualised costs of rolling out surgical sterilisation-immunization, injectable immunization and oral immunizations were estimated to be $ 44 million (Rs 2,350 million), $23 million (Rs 1,230 million) and $ 11 million (Rs 590 million), respectively. Dog bite incidence, health systems coverage and cost of rabies biologicals were found to be important drivers of costs for human interventions. For the animal sector interventions, the size of dog catching team, dog population and vaccine costs were found to be driving the costs. Rabies control in Tamil Nadu seems a costly proposition the way it is currently structured. Policy makers in Tamil Nadu and other similar settings should consider the long-term financial sustainability before embarking upon a state or nation-wide rabies control programme.

## Author Summary

Rabies is a fatal viral disease. It is transmitted mostly through dog bites in greater parts of Asia and Africa. It is primarily a disease of the poorer population groups with children being the most vulnerable. Control of rabies among humans therefore requires interventions in the animal as well as the human sectors. Animal sector interventions include vaccination accompanied with or without sterilization of dogs. Human interventions are limited to individual vaccination following dog bites. We estimated the costs to the government of rolling out animal as well as human sector interventions across an entire state having a human population of 72 million. We also estimated the major drivers influencing program costs and the implications to the government of adopting such a strategy over a long time. We found that the animal sector interventions were many times more costly than the most expensive human interventions. We also found that in the absence of dog population control measures, it will require substantial financial commitment on the part of the government to be able to invest in dog vaccination strategies.

## Introduction

### Background & objective

While rabies has been identified as a priority zoonoses that needs to be addressed globally [1], it has a special relevance in South Asia. More than 55,000 rabies deaths have been estimated to occur among humans annually with little under half being contributed by India alone [2], [3]. Experts from animal as well as human health sectors agree on the controllable nature of the disease and on the importance of joint population level interventions for restricting disease transmission among animals and humans [4], [5].

### Knowledge gaps

Evidence from India and elsewhere demonstrates the efficacy of principle rabies intervention strategies. Indian researchers have studied the application of different post-exposure prophylaxis (PEP) regimens among humans [6]. Indian researchers have also used the experience of dog population control in specific urban settings to demonstrate the impacts of animal birth control strategies [7], [8]. Of late there is mounting evidence produced by international researchers related to the efficacy of anti-rabies immunization among animals in reducing rabies transmission [9]. Economic assessments have also been conducted in different parts of the world which study the economic impact of rabies [2], economics of rabies control [10] and cost effectiveness of different post-exposure prophylaxis regimens [11]. This body of work has been instrumental in development of national strategic plans for rabies control [12].

However, as previously documented, rabies researchers have not been able to satisfy the information needs of policymakers [13] and the economics of rabies control remains a “significant constraint” in rolling out rabies control programmes in low income countries [14], [15]. A possible explanation could be that to date, only a handful of studies have looked at combined costs of rabies across human and animal sectors [2], [10], [16]. Most of these analyses have been conducted from the societal perspective that is of limited use to program managers. Additionally, because of the design of cost effectiveness analyses, their findings are always relative in nature and are difficult to generalise in absolute terms.

Accordingly, we undertook a costing exercise building upon an earlier assessment [17] of rabies control initiative in the Southern Indian state of Tamil Nadu. Its objective was to determine the costs to the government of implementing different combinations of strategies for controlling rabies among human and animal populations in a state like Tamil Nadu.

### Tamil Nadu rabies control initiative

Tamil Nadu is the southernmost state in India having a population of 72 million [18] and is considered one of the better performing states in public health [19]. According to the results of a study based upon verbal autopsy of deaths between 2001–03, it had 0.5 deaths or fewer per 100,000 human population due to furious rabies [20]. In response to calls for controlling dog bites and rabies, the state government formed a state level rabies coordination committee in 2008 to develop and manage a multisectoral response to dog bites and rabies in the state. This was the first time a large scale population level rabies control intervention was implemented in a large state in India [17].

As described in Table 1, the human interventions consisted of ensuring availability of anti-rabies vaccine at all government-run health facilities in the state as well as promoting awareness about rabies control across the state. Rabies antibody was not provided universally due to perceived high costs. The animal interventions involved outsourcing of ABC-AR operations to private veterinarians; dog catching operations were handled by local animal welfare organizations in selected urban areas of the state. ABC-AR was conducted throughout the year as specified in the guidelines of Animal Welfare Board of India [21]; vaccination-only strategies, whether parenteral or oral, were not considered. The interventions were supposed to be implemented in a continuous fashion throughout the year and not conducted in a campaign mode. The animal and human sector interventions were implemented by different departments and coordinated at the state and district levels through formal multi-stakeholder coordination mechanisms [17].

**Table 1:** Interventions employed for rabies control in Tamil Nadu.

Implementing Sector	Interventions for rabies control	Included in costing framework
**Animal interventions**	Laws enacted for licensing of dogs	No
	Animal population census (annual)*	Yes+
	Animal Birth Control – Anti Rabies Vaccination**	Yes++
	Training of dog handlers	Yes+
	Community awareness	Yes+
**Human Interventions**	Inclusion of dog bite cases in disease surveillance system	No
	Easy availability of anti-rabies vaccination	Yes+
	Training for intra-dermal vaccination**	Yes+
	Antibody provision***	Yes++
	Community Awareness	Yes+
**Systemic interventions**	Establishment of intersectoral coordination mechanisms	No
	Procurement and Supply Chain Management System	No
	Waste disposal system in urban municipalities	No

* Annual census done in pilot urban municipalities only, Livestock census done every four years in other areas.

** ABC-AR implemented in pilot urban municipalities only.

*** Antibody provision in selected districts only.

+ Included in costing of all combination of interventions.

++ Included in costing of selected combination of interventions only.

## Methods

Using program data from the earlier assessment in Tamil Nadu, we estimated the annual costs of scaling up those interventions across the state, including rural areas. An activity based costing approach was used. The interventions for human and animal populations were calculated separately and the costs for different components within these interventions were disaggregated. System-wide and environmental interventions, such as waste management and vaccine supply chain management systems were not included in the costing framework (Table 1). Costs were calculated from the perspective of government, which was the provider for bulk of the services. Costs were estimated for five different combinations of interventions described in Table 1. All costs were inflated to 2012 Indian Rupees using national financial data [22] and converted into 2012 US dollars using historical exchange rates [23].

### Human sector interventions

Based upon the existing interventions in Tamil Nadu [17], it was assumed that the entire population (rural as well as urban) would be covered by the expanded intervention. Costs were estimated for two combinations of interventions. Based upon the existing intervention model, the first set of interventions consisted of increased surveillance and awareness, in addition to provision of anti-rabies vaccine (ARV) to all patients reporting dog bites at public health facilities. The second combination of interventions involved an additional component of antibody administration to patients with severe dog bites in addition to the ARV.

Based upon the feedback received from local program managers [24], it was assumed that dog bite cases that report at peripherally located and low-throughput health centres would be provided with rabies vaccine through the easier intramuscular route, while those that report at high-throughput hospitals with better trained personnel would be provided vaccination through the intradermal route. The procurement costs of intradermal and intramuscular vaccine formulations (having different vial sizes) and antibodies were estimated from the state level procurement records [25] and market data, respectively. A standard 30% wastage rate was assumed for both the vaccine formulations in the absence of specific reference points. A lesser wastage rate of 15% was used for the antibody.

The annual number of outpatient visits for dog bites was calculated from the monthly dog bite visits reported by the state disease surveillance system over a twenty month period from January 2008 to August 2009. This was divided by the expected number of hospital visits for each dog bite case, to arrive at the annual number of dog bites in the state. While the national guidelines [26] recommend vaccination only for category 2 and category 3 dog bites, in practice, the vaccine was being administered to all reported dog bite cases, which was factored into our analysis. The proportion of dog bites categorised as ‘severe’ and requiring antibodies was assumed to be 63%, using estimates from other national studies [27]. Based upon the feedback received from program managers, some program administration costs were included to cover expenditure related to awareness generation, training and surveillance related activities.

### Animal sector interventions

The then-prevalent model of ABC-AR was selected as one of the intervention strategies. Parenteral vaccination using teams of dog-catchers and oral vaccination were selected as hypothetical intervention scenarios to determine the extent to which costs could be reduced by less resource-intensive exercises.

Using dog population density figures from the livestock census [28], the number of animal sheds (having capacity for 30–45 animals) required to cater to 100,000 human populations were calculated. The fixed and recurrent costs were then calculated for every 100,000 population. The costs for animal interventions were sourced from state program guidelines and adjusted for inflation. Subsequently, differential costing was conducted to include additional stay and veterinary fees for operating on female dogs. More vehicles were assumed to be required in rural areas because of the larger distances to be covered. Therefore, increased capital and fuel costs were considered for dog shelters in rural areas. All capital costs were depreciated over 5 years. Costing for dog catchers' and ambulance drivers' time was done on a monthly basis using state salary norms. Animal census costs were also included as an annual exercise and estimated accordingly.

### Senstitivity analysis

A base case scenario was constructed for each of the five different combinations of human and animal interventions using the existing or most likely estimates of key input parameters. The values of input data for our analysis were sourced from our review of program documents, published research literature and from our personal observations in the state. A sensitivity analysis was conducted by varying the values of principle input factors. More than 224,000 scenarios of animal and human interventions were tested. The values of input parameters for the base case and alternative scenarios have been described in Supplementary Files. These were refined based upon the feedback received from experts at two different national consultations of Indian rabies experts organized in 2011 [29] and 2013 [30].

### Projected costs

Rabies control is a long term proposition, requiring sustained levels of high coverage of interventions in the animal populations [10]. Accordingly, in addition to estimating the annual costs on the basis of a one-time assessment, we also assessed the long term implications of the animal sector interventions. Given the limited data on the impact of parenteral animal vaccination campaigns in mixed ecological settings such as India, we used data from an Indian study [7] describing the impact of dog population management interventions to assess the long term implications of the animal sector interventions.

We projected costs of four interventions—ABC-AR, injectable vaccination, oral vaccination, and a hypothetical intervention coupling injectable vaccination with injectable contraception for 20 years based on 2012 costs. For interventions involving contraception, a decrease in the dog population was estimated from a dog demographic model used earlier in India [7]. The model estimated the change in total stray dog population and the proportion of sterile dogs over a 20-year period given a sterilization rate of 62–87% in several mark-recapture study areas in Jodhpur city, from 2005 to 2007.

Since no other dog demographic models in the Indian context were available, we estimated the total number of stray dogs and its proportion that would be sterile for each year in Tamil Nadu assuming a similar setting and level of coverage. To project costs for future years, annualized capital costs (for 5-year depreciation) were assumed to be constant over 20 years, and recurrent costs were scaled to the projected dog population size in each year. Recurrent costs were calculated separately for the unsterile (requiring vaccination and sterilization) and sterile (requiring only vaccination) dog populations. Interventions which did not involve sterilization assumed a constant dog population. For the hypothetical injectable vaccination and contraception intervention, the additional cost of the injectable contraceptive was assumed to be negligible and the initial cost in 2012 was assumed to be the same as the cost of the injectable vaccine intervention alone in the base case scenario.

### Limitations

The study is based upon one-time costs data collected from state programme managers. Therefore the analysis only considers those human cases that were reported to the public health surveillance system. This is likely to be an underestimate. Moreover, there is limited data on the completion of treatment; and it is possible that a small portion of patients might not complete their treatment, leading to a further underestimate of dog bite incidence rate. Data on categorization of dog bites, dog bite burden among animals and dog ecology is limited. In the absence of more data, the upper and lower bounds of the input parameters were taken from a range of sources, including expert opinion, summarised in Supplementary Files 1 & 2. In the absence of longitudinal data, we used dog demographic projections from an Indian study [7] to estimate the long term resource requirements for different rabies control interventions. However, there is limited information related to the reliability of these findings in rural areas and other parts of India. While recent studies recommend canine vaccination in annual campaigns having coverage exceeding 60%[9], the current analysis estimates the cost of an year-long continuous routine vaccination strategy which is likely to provide a conservative estimate of likely costs. More long-term efficacy studies for different interventions are required to better comment upon their cost effectiveness.

## Results

### Base case scenario

The annual costs of providing post exposure prophylaxis with antibodies for severe dog bites for Tamil Nadu was calculated to be $ 2.2 million (Table 2). This was more than three times the costs of rolling out a vaccine-only program and translates into costs of $ 11 and $ 3, respectively for each dog bite patient vaccinated. Using base case scenarios, the annual costs of implementing ABC-AR, Injectable vaccination and oral vaccine programmes were calculated to be $ 44 million, $ 23 million and $ 11 million, resulting in each dog's vaccination costing $ 22, $ 11 and $ 5, respectively.

**Table 2:** Total annual costs of implementing human and animal rabies control interventions in Tamil Nadu in 2012.

Interventions	Total annual program costs	Cost per dog bite patient/Cost per vaccinated dog	Cost per capita
**Human Interventions**			
Anti-rabies vaccine+Antibodies	$ 2.2 million (Rs. 119 million)	$ 11 (Rs. 607)	$ 0.03 (Rs. 1.6)
Anti-rabies vaccine only	$ 0.7 million (Rs. 36 million)	$3 (Rs. 185)	$ 0.01 (Rs. 0.5)
**Animal Interventions**			
Surgical Animal Birth Control+Vaccinations	$ 44 million (Rs. 2,350 million)	$ 22 (Rs. 1,164)	$ 0.6 (Rs. 33)
Injectable Vaccinations only	$ 23 million (Rs. 1,230 million)	$ 11 (Rs. 607)	$ 0.3 (Rs. 17)
Oral Vaccinations only	$ 11 million (Rs. 590 million)	$ 5 (Rs. 290)	$ 0.2 (Rs. 8)

### Sensitivity analysis

**Figure 1:**
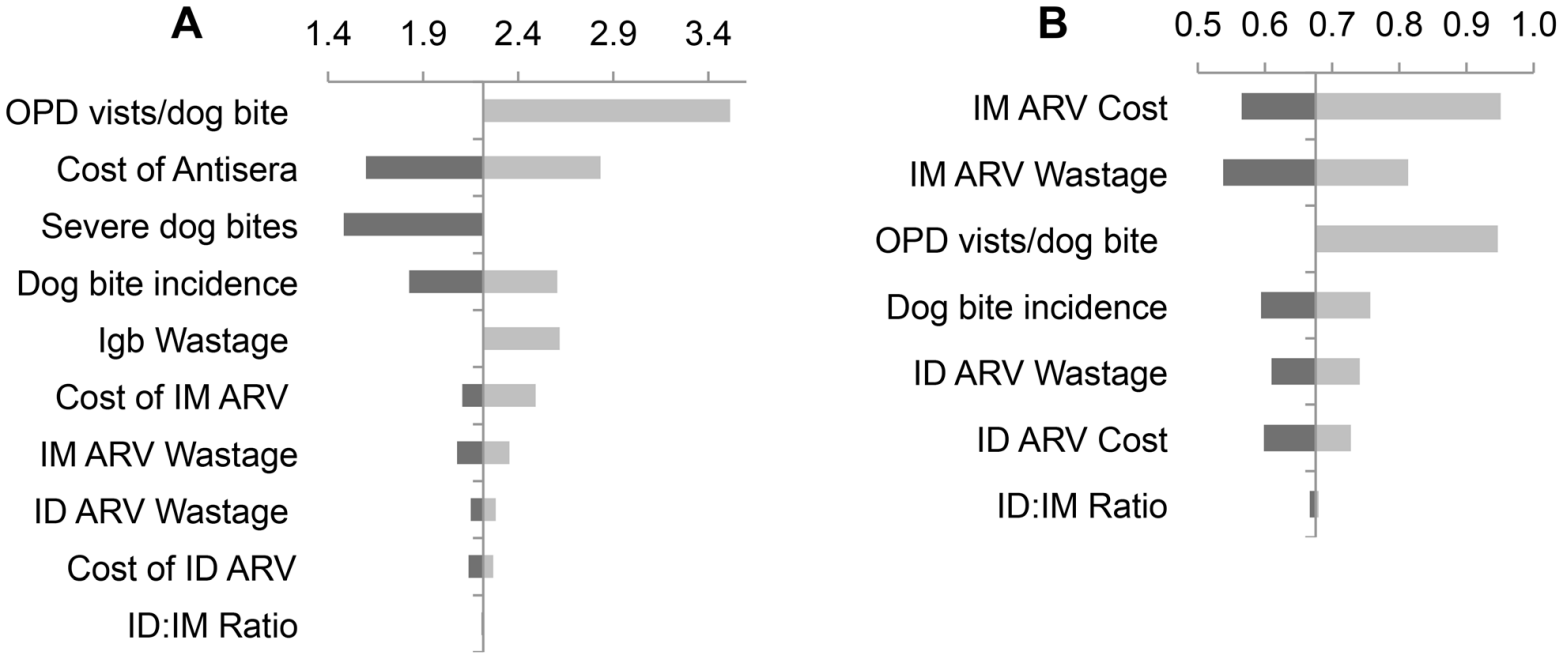
Cost drivers for state-wide human rabies interventions (in 2012 Million US$): A. Antirabies virus and Antibody immunization program; B. Antirabies vaccine only program (Y axis represents intervention costs for base case scenario).

**Figure 2:**
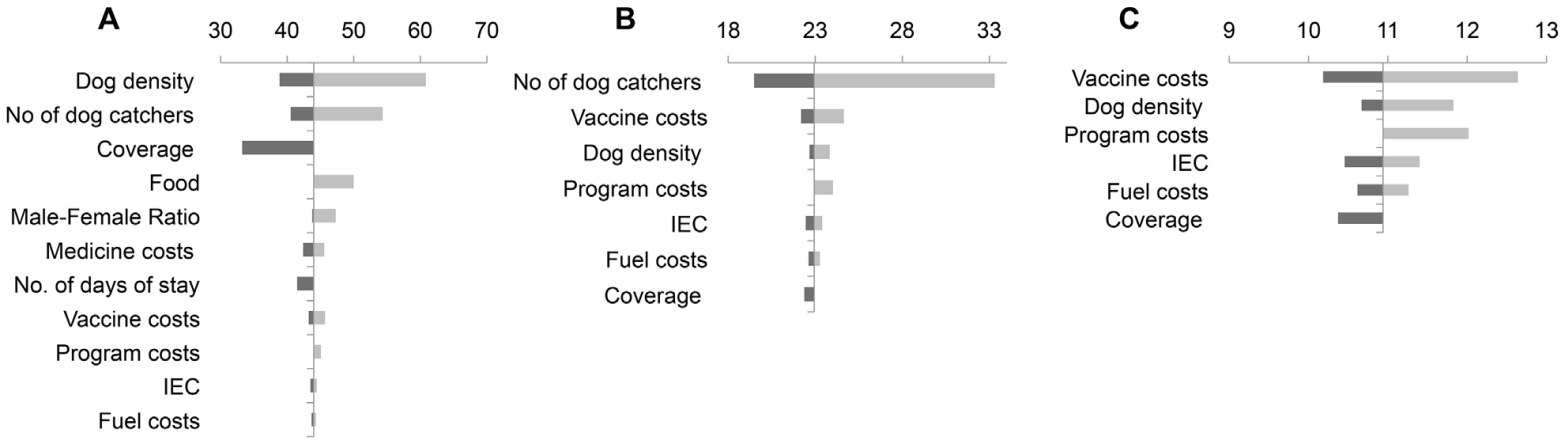
Cost drivers for state-wide animal rabies interventions (in 2012 Million US$): A. Canine Animal Birth Control & Immunization program; B. Canine Injectable vaccination program; C. Canine Oral vaccination programme (Y axis represents intervention costs for Base Case Scenario).

On varying the key input parameters, we found that the costs of the human interventions ranged from $3–$82 million, while the costs for animal intervention ranged from $9–$98 million annually. In order to compare the relative effects of different cost components on individual set of interventions, a tornado chart was prepared (Figure 1 & Figure 2) centred around the costs of the base case scenario for each combination of interventions. The value of each cost component was varied to its upper and lower bounds and the impact on the total program cost charted as red and blue bars, respectively.

In the case of human interventions (Vaccine only and Vaccine+Antibodies), health seeking patterns, cost and wastage rates of vaccine and antisera and the burden of dog bites were found to be the major cost drivers causing the greatest fluctuations in the cost of the program. In relation to the other drivers, the use of intradermal versus intramuscular vaccine regimes did not greatly influence program costs. Antibody procurement comprised around 70% of total costs of the human sector interventions, followed by vaccine procurement costs and training & health promotion costs.

In case of animal-sector interventions, the dog population and number of dog catchers required per team appear to be important drivers of ABC-AR programme costs. On the other hand, vaccine costs have greater role to play in influencing costs of vaccination-only programmes. Sex distribution of dogs does not affect total program costs in the long term, even for ABC-AR in which different surgical procedures are required for male and female dogs.

### Projections

Assuming an average sterilization rate of 62–87%, the dog population size in Tamil Nadu would be expected to decrease by 70% over a 20 year period from an estimated 2,022,055 dogs in 2012 to 615,408 in 2032 (Supplementary File 4). The proportion of sterilized dogs would stabilize at 80% such that the number of dogs needing ABC would decrease by 94% from 2,022,055 in 2012 to 123,082 dogs in 2032.

**Figure 3:**
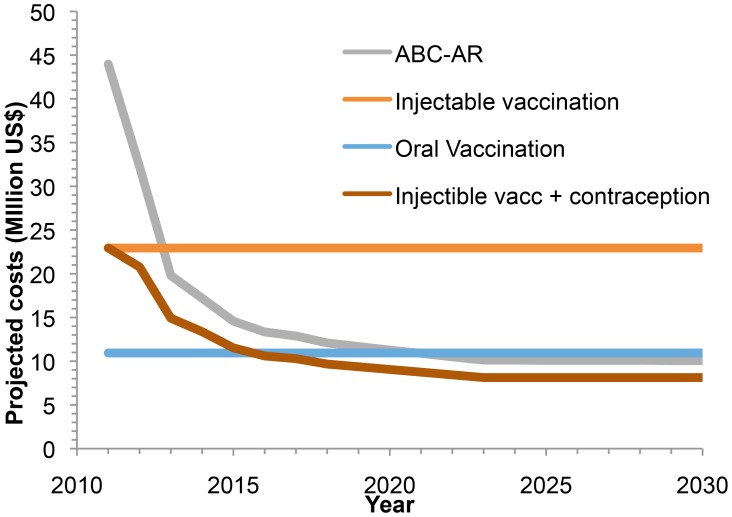
Projected costs (in million US$) from 2012 to 2032 for four different animal sector interventions for rabies control in Tamil Nadu: ABC-AR; injectable vaccination; oral vaccination; and injectable vaccination cum contraception.

Projected costs for ABC-AR, injectable vaccination, oral vaccination, and injectable vaccination-cum-contraception are shown in Figure 3. While costs are highest for ABC-AR in 2012, the cost drops quickly and is lower than that for injectable vaccination and similar to that for oral vaccination by 2032 (Table 3). Total costs over the 20-year period are highest for injectable vaccination and are comparable for oral vaccination and ABC-AR. The hypothetical joint injectable vaccination and contraception intervention would result in the lowest cost by 2032 and the lowest total cost.

**Table 3:** 20 year cost projections and total cost for ABC-AR, injectable vaccination, oral vaccination, and injectable vaccination and contraception animal rabies interventions in Tamil Nadu.

Intervention	Program costs in 2012	Program costs in 2032	Total cost (2012–2032)
**ABC-AR**	$ 44 million (Rs. 2,354 million)	$ 10 million (Rs. 539 million)	$ 301 million (Rs. 16,128 million)
**Injectable vaccination**	$ 23 million (Rs. 1,228 million)	$ 23 million (Rs. 1,228 million)	$ 482 million (Rs. 25,792 million)
**Oral vaccination**	$ 11 million (Rs. 586 million)	$ 11 million (Rs. 586 million)	$ 230 million (Rs. 12,298 million)
**Injectable vaccination and contraception**	$ 23 million (Rs. 1,228 million)	$ 28 million (Rs. 435 million)	$ 223 million (Rs. 11,931 million)

## Discussion

In keeping with assertions about rabies control being the responsibility of the local governments [31], this study was conducted from the perspective of the state government of Tamil Nadu to inform its efforts to control rabies in the state. While by no means definitive, these results help in identifying major drivers of costs within a range of government sponsored intervention strategies.

The identification of major drivers of programme costs can inform programme management by identifying areas to improve program efficiency and direct research efforts towards development of precise estimates where required. Basic epidemiological parameters such as incidence of dog bites, their categorization and dog population density were found to be among the major drivers of the program costs. These knowledge gaps require more attention from researchers and can be easily filled through focussed research studies. From the program perspective, the procurement and wastage rates of vaccine and antibodies were found to greatly influence total program costs. Strengthening of local procurement and supply chain management systems and negotiating long-term procurement rates are some of the options that could help offset these costs.

### Selecting animal interventions

Using base case scenarios, scaling up the existing animal interventions (ABC-AR) in Tamil Nadu would require 20 to 65 times the funds required for scaling up human post exposure prophylaxis alone. Moreover, a combination of human Post-exposure prophylaxis with ABC-AR would cost over 2.1% of the annual budgetary allocations for the departments of health, animal husbandry and municipal administration together in Tamil Nadu [32].

This is an important lesson for the proposed national rabies control programme in India which is currently structured around financing ABC-AR operations across selected cities [33]. Recent discussions have advocated parenteral vaccination of canines as a first step towards elimination of rabies [1], [9]. This would require a high level of coverage (>60%) costing 27% of the annual budget of the state department of animal husbandry, the likely implementing agency for such an intervention, and would need to be sustained continuously for multiple years or even decades.

Due to the challenges of achieving high vaccination coverage even among humans [34] and the high costs of existing animal interventions described above, the policymakers are unlikely to commit to a comprehensive rabies control programme yet. A more favourable case for rabies control among canines could be made by developing newer animal interventions that are not only efficacious but also affordable and effective, such as an inexpensive canine injectable contraceptive cum vaccine. In the absence of an intervention that promises long term sustainability, it is likely that ad hoc measures like post exposure vaccinations to economically productive animals continue.

### Selecting human interventions

Antibodies were not made universally available for human vaccines because of the costs involved. Our calculations show that the costs of a combined (antibody plus vaccine) rabies programme would be three times the costs of vaccine only intervention and is likely to cost an additional expenditure of $ 1.5 million (Rs. 82.5 million) annually.

Given the costs of different vaccine formulations in Tamil Nadu, choosing intradermal over intramuscular vaccine regimen is likely to result in annual savings of $ 13,000 (Rs 700,000) only. This relatively small amount should not be a deterrent to state public health programme managers in choosing a vaccine regimen that is more appropriate to the clinical setting and qualifications of their staff [24].

### Conclusions

Rabies control efforts in Tamil Nadu seem a costly proposition as they are currently structured in the state. This would necessarily require high levels of technical, political and financial commitments before the government chooses to embark upon a long-term rabies control strategy. Given recent recognition of the need for a national rabies control programme in India by the National Centre for Disease Control [33] and the FAO/WHO/OIE tripartite statement on inclusion of rabies as an ‘entry point’ for demonstrating zoonoses control efforts at the global level [1], it is important that these discussions adopt a long term perspective and take local complexities into account before developing a national or a global rabies elimination strategy.

## Supporting Information

Supporting Information S1 Input factors used for the cost analysis of Human Interventions.
(DOCX)

Supporting Information S2 Input factors used for the cost analysis of Animal Interventions.
(DOCX)

Supporting Information S3 Breakup of animal intervention costs.
(DOCX)

Supporting Information S4 Program-adjusted rates of ABC-AR.
(DOCX)

Supporting Information S5 Dog demographic model.
(DOCX)

Supporting Information S6 Calculations.
(XLSX)
