# Supplementary material for: Costs Analysis of a Population Level Rabies Control Programme in Tamil Nadu, India
Source: PLoS Negl Trop Dis. 2014 Feb 27;8(2):e2721. doi: 10.1371/journal.pntd.0002721 (PMC3937306; doi:10.1371/journal.pntd.0002721)
Supplement: Supporting Information S1 — Input factors used for the cost analysis of Human Interventions. (DOCX) [file pntd.0002721.s001.docx]

# Supporting Information File S1: Input factors used for the cost analysis of Human Interventions

| Cost heads | Base Case | Alternative Scenarios | Source of information |
| --- | --- | --- | --- |
| Dog bite OPD vists (per lakh population) | 1356 | 1085 / 1627 | Base case value from surveillance data[1]; alternative values calculated as +/- 20% |
| Number of OPD visits per dog bite | 5 | 3 | Feedback received from state program officers [2] |
| ID to IM Use ratio | 0.33 | 0 / 0.66 / 1 | Base case value based upon distribution of dog bites in high throughput to low throughput facilities, as reported by different directorates[2] |
| Vaccine required per dog bite case in ml (ID) | 1 |  | Updated Thai Red Cross schedule as mentioned in national guidelines [3] |
| Vaccine required per dog bite case in ml (IM) | 5 |  | National guidelines [3] |
| Wastage Rates for ARV (ID & IM) | 30% | 15% / 45% | Assumption |
| Cost of procuring 1 ml of ARV (IM) in Rs | 75 | 45 / 105/ 150 | Based upon vaccine procurement data & Expert opinion |
| Cost of procuring 1 ml of ARV (ID) in Rs | 363 | 218 / 508 / 150 | Based upon vaccine procurement data & Expert opinion |
| Category 3 dog bites as a proportion of total dog bites | 63% | 33% / 50% | National Study [4] |
| Cost of procuring 1500 IU of antisera in Rs | 582 | 349 / 815 | Expert opinion |
| Wastage rate for antibody | 15% | 30% / 45% | Assumption |
| Incremental program costs per district (Training + IEC + Surveillance + Monitoring & Supervision) in Rs | Rs 436,000 |  | Program documents [2] & Assumptions |

# References

1. Abbas SS, Venkataramanan V, Kakkar M, Pathak G (2011) Rabies control initiative in Tamil Nadu, India: a test case for the “One Health” approach. International Health 3: 231–239. doi:10.1016/j.inhe.2011.08.001.

2. Kakkar M, Abbas SS, Pathak G (2009) Rabies Control Initiative in Tamil Nadu. New Delhi.

3. National Institute of Communicable Diseases (2007) National Guidelines for Rabies Prophylaxis and Intra-dermal Administration of Cell Culture Rabies Vaccines. Available: http://www.nicd.nic.in/ncdc_new/Rabies_Guidelines.pdf. Accessed 2 April 2011.

4. Ichhpujani RL, Mala C, Veena M, Singh J, Bhardwaj M, et al. (2008) Epidemiology of animal bites and rabies cases in India. A multicentric study. The Journal of communicable diseases 40: 27–36.
