# Supplementary material for: Costs Analysis of a Population Level Rabies Control Programme in Tamil Nadu, India
Source: PLoS Negl Trop Dis. 2014 Feb 27;8(2):e2721. doi: 10.1371/journal.pntd.0002721 (PMC3937306; doi:10.1371/journal.pntd.0002721)
Supplement: Supporting Information S2 — Input factors used for the cost analysis of Animal Interventions. (DOCX) [file pntd.0002721.s002.docx]

# Supporting Information File S2: Input factors used for the cost analysis of Animal Interventions

| Cost heads | Base Case | Alternative Scenarios | Source of information |
| --- | --- | --- | --- |
| Average TN dog density (per lakh humans) | 2803 | 2135 / 2803 / 5000 | State animal census [1–3] |
| Dog Male-Female Ratio in Tamil Nadu | 2.62 | 0.5 / 1 / 2 / 3 | Livestock census (2004) |
| Oral/Injectible Vaccine costs per dog (plus wastage) in Rs | 30 | 10 / 75 | Program managers |
| Length of stay for male dogs for ABC (days) | 4 | 1 / 2 | Program managers |
| Length of stay for female dogs for ABC (days) | 7 | 1 / 2 | Program managers |
| Number of dog catchers per team | 3 | 2 / 6 | Program managers |
| Incremental costs of training per lakh population in Rs | 20,000 | 50,000 / 1,00,000 | Program data, Assumption |
| Incremental costs of IEC per lakh population in Rs | 85,882 | 50,000 / 1,20,000 | Program data, Assumption |
| Coverage of interventions | 100% | 50% / 70% | Assumption |
| Dog enumeration / survey costs per 100,000 population in Rs | 50,000 |  | Assumption |
| Incremental costs of procuring dog catching vehicles in Rs | 4,00,000 |  | Assumption |
| Incremental costs for renovating animal shed for ABC in Rs | 2,33,288 |  | Program data, inflation adjusted |
| Cost of dog catching equipment | 3,435 |  | Program data, inflation adjusted |
| Ambulance running costs per KM | 6 | 5 / 7 | Program data, inflation adjusted |
| Daily distance to be covered per ambulance in urban areas (kms) | 50 |  | Assumption |
| Daily distance to be covered per ambulance in rural areas (kms) | 100 |  | Assumption |
| Monthly salary of one driver in Rs | 17,176 |  | Program data, inflation adjusted |
| Monthly salary of one dog catcher in Rs | 14,085 |  | Program data, inflation adjusted |
| Vet expenses per male dog in Rs | 103 |  | Program data, inflation adjusted |
| Vet expenses per female dog in Rs | 172 |  | Program data, inflation adjusted |
| Paravet expenses per male dog in Rs | 86 |  | Program data, inflation adjusted |
| Paravet expenses per female dog in Rs | 103 |  | Program data, inflation adjusted |
| Medicine costs per male dogs for ABC-AR in Rs | 185 |  | Program data, inflation adjusted |
| Medicine costs per male dogs for ABC-AR in Rs | 271 |  | Program data, inflation adjusted |
| Other costs per operated dog in Rs | 34 |  | Program data, inflation adjusted |

## References

1. Department of Animal Husbandry Darying And Fisheries (2004) 17th Livestock Census, 2003. New Delhi.

2. Department of Animal Husbandry Darying And Fisheries (2009) 18th Livestock Census, 2007. New Delhi.

3. Commissioner of Municipal Administration - Government of Tamil Nadu (2007) Implementation of Animal Birth Control Program in 5 Corporations and 50 Municipalities - G.O. (MS) No.10, dated 18.1.2007: 4.
