# Supplementary material for: Costs Analysis of a Population Level Rabies Control Programme in Tamil Nadu, India
Source: PLoS Negl Trop Dis. 2014 Feb 27;8(2):e2721. doi: 10.1371/journal.pntd.0002721 (PMC3937306; doi:10.1371/journal.pntd.0002721)
Supplement: Supporting Information S3 — Breakup of animal intervention costs. (DOCX) [file pntd.0002721.s003.docx]

# Supporting Information File S3: Breakup of animal intervention costs (2012 US$ million)

| Cost centre | ABC-AR | AR (Inj) | AR (Oral) |
| --- | --- | --- | --- |
| Fuel | 3.2 | 3.2 | 3.2 |
| Driver | 4.2 | 4.2 | 4.2 |
| Dog catcher | 10.4 | 10.4 | - |
| Veterinarian fees | 4.6 | - | - |
| Paraveterinarian fees | 3.4 | - | - |
| Medicines | 7.9 | - | - |
| Parenteral rabies vaccine | 1.1 | 1.1 | 1.1 |
| Food | 3.1 | - | - |
| Miscellaneous | 1.3 | - | - |
| Utilities | 0.3 | 0.3 | 0.3 |
| Training | 0.3 | 0.3 | 0.3 |
| Awareness & IEC | 1.2 | 1.2 | 1.2 |
| Annual census | 0.7 | 0.7 | 0.7 |
| Annualised capital cost | 2.3 | 1.6 | - |
| TOTAL | 44.0 | 22.9 | 10.9 |
