# Supplementary material for: Costs Analysis of a Population Level Rabies Control Programme in Tamil Nadu, India
Source: PLoS Negl Trop Dis. 2014 Feb 27;8(2):e2721. doi: 10.1371/journal.pntd.0002721 (PMC3937306; doi:10.1371/journal.pntd.0002721)
Supplement: Supporting Information S4 — Program-adjusted rates of ABC-AR. (DOCX) [file pntd.0002721.s004.docx]

# Supporting Information File S4: Program-adjusted rates of ABC-AR

| Cost Component | TN Govt rates (2006) | Inflation adjusted rates (2012) | Program adjusted rates (2012) |
| --- | --- | --- | --- |
| Para vets fees | $0.93 (Rs. 50) | $1.6 (Rs. 86) | $1.69 (Rs. 91) |
| Dog catcher salaries | $0.47 (Rs. 25) | $0.8 (Rs. 43) | $5.12 (Rs. 274) |
| Surgeon fees | $1.12 (Rs. 60) | $1.93 (Rs. 103) | $2.28 (Rs. 122) |
| Vaccine and Medicines | $3.18 (Rs. 170) | $5.45 (Rs. 292) | $4.45 (Rs. 238) |
| Food for admitted dogs | $1.31 (Rs. 70) | $2.25 (Rs. 120) | $1.55 (Rs. 83) |
| Ambulance and driver costs | $0.93 (Rs. 50) | $1.6 (Rs. 86) | $3.67 (Rs. 197) |
| Miscellaneous expenses | $0.37 (Rs. 20) | $0.64 (Rs. 34) | $0.64 (Rs. 34) |
| Utilities |  |  | $0.64 (Rs. 7) |
| Training |  |  | $0.14 (Rs. 7) |
| IEC |  |  | $0.13 (Rs. 31) |
| Census |  |  | $0.57 (Rs. 18) |
| Annualised capital costs |  |  | $0.33 (Rs. 62) |
| Total amount per dog | $8.31 (Rs. 445) | $14.28 (Rs. 764) | $21.23 (Rs. 1164) |
