# Supplementary material for: Costs Analysis of a Population Level Rabies Control Programme in Tamil Nadu, India
Source: PLoS Negl Trop Dis. 2014 Feb 27;8(2):e2721. doi: 10.1371/journal.pntd.0002721 (PMC3937306; doi:10.1371/journal.pntd.0002721)
Supplement: Supporting Information S5 — Dog demographic model. (DOCX) [file pntd.0002721.s005.docx]

# Supporting Information File S5: Dog demographic model

Dog demographic model estimating the decrease in dog population in Tamil Nadu over 20 years as a result of 62-87% coverage of ABC. Blue diamonds indicate total number of stray dogs, and red squares indicate the number of unsterile stray dogs needing ABC based on the proportion of the dog population which has been sterilized. Model is adapted from Totton et al[1].

# References

1. Totton SC, Wandeler AI, Zinsstag J, Bauch CT, Ribble CS, et al. (2010) Stray dog population demographics in Jodhpur, India following a population control/rabies vaccination program. Preventive veterinary medicine 97: 51–57. doi:10.1016/j.prevetmed.2010.07.009.
